# Imaging β-Cell Function Using a Zinc-Responsive MRI Contrast Agent May Identify First Responder Islets

**DOI:** 10.3389/fendo.2021.809867

**Published:** 2022-01-31

**Authors:** Bibek Thapa, Eul Hyun Suh, Daniel Parrott, Pooyan Khalighinejad, Gaurav Sharma, Sara Chirayil, A. Dean Sherry

**Affiliations:** ^1^ Advanced Imaging Research Center, The University of Texas Southwestern Medical Center, Dallas, TX, United States; ^2^ Department of Radiology, The University of Texas Southwestern Medical Center, Dallas, TX, United States; ^3^ Department of Chemistry and Biochemistry, The University of Texas at Dallas, Richardson, TX, United States

**Keywords:** metabolic imaging, zinc-responsive contrast agent, glucose-stimulated insulin secretion (GSIS), glucose stimulated zinc secretion (GSZS), magnetic resonance imaging, pancreatic β-cell function

## Abstract

An imaging method for detecting β-cell function in real-time in the rodent pancreas could provide new insights into the biological mechanisms involving loss of β-cell function during development of type 2 diabetes and for testing of new drugs designed to modulate insulin secretion. In this study, we used a zinc-responsive MRI contrast agent and an optimized 2D MRI method to show that glucose stimulated insulin and zinc secretion can be detected as functionally active “hot spots” in the tail of the rat pancreas. A comparison of functional images with histological markers show that insulin and zinc secretion does not occur uniformly among all pancreatic islets but rather that some islets respond rapidly to an increase in glucose while others remain silent. Zinc and insulin secretion was shown to be altered in streptozotocin and exenatide treated rats thereby verifying that this simple MRI technique is responsive to changes in β-cell function.

## Introduction

The islets of Langerhans are functional units of the endocrine pancreas consisting of at least five different cell types: α-cells, β-cells, δ-cells, F-cells, and ϵ-cells (1). The most highly abundant are β-cells, which synthesize, store, and secret insulin in response to post-prandial increases in blood glucose (2). Given their role in regulation of glucose metabolism, β-cells are pivotal in the pathogenesis of diabetes mellitus (DM), a metabolic disorder characterized by insulin dysregulation and chronic hyperglycemia (3). Broadly speaking, there are two main types of diabetes (4); Type 1 DM (T1DM) is characterized by autoimmune destruction of β-cells leading to insulin deficiency and metabolic disease (5, 6) while type 2 DM (T2DM) is characterized by a combined loss of β-cell function and peripheral insulin resistance. Nearly 462 million individuals, or 6.28% of the world’s population, are affected by T2DM with more than 1 million deaths annually, ranking T2DM the ninth leading cause of mortality (7, 8). The first clinical indication of T2DM is insulin resistance-induced hyperglycemia (IRIH) characterized by decreased responsiveness of peripheral cells to insulin (9). To compensate for IRIH, β-cells secret increasing amounts of insulin as the disease progresses until the mass of β-cells begins to decline and undergo apoptosis (10). As glucose-stimulated insulin secretion (GSIS) is a hallmark of normal β-cell function, understanding the pathological steps that lead to its impairment is a critical challenge.

Pancreatic β-cells have an exceptionally high zinc (Zn^2+^) content which is essential for synthesis, structural stability, and storage of insulin (11). Insulin is stored in pancreatic β-cell granules as a crystalline hexamer containing two Zn^2+^ ions and one calcium (Ca^2+^) ion (12, 13). Upon stimulation of β-cells by secretagogues (most often, glucose), Zn^2+^ is co-released with insulin and this increases the Zn^2+^ concentration in the extracellular space (ECS) to more than 10-fold higher than the basal serum level (~40 μM basal to ~500 μM after secretion) (14). Given co-release of insulin and Zn^2+^ by β-cells, a method for imaging Zn^2+^ secretion in real-time could improve pathophysiologic understanding of the development of T2DM. Several independent studies have reported that zinc supplementation potentiates the oxidative stress caused by hyperglycemia and ameliorates glucose, HbA1c, and lipid levels in diabetic patients (11, 15). This suggests that functional imaging of pancreatic β-cells, especially at the level of individual islets could be used to better understand the pathophysiology of impaired GSIS.

In addition to optical methods, several other imaging modalities have been used to image β-cell mass (16 *–* 17) and function (18, 19) *in vivo* in rodents. Although the rodent model is widely used, there are technical challenges in imaging the rodent pancreas because, unlike the human pancreas, the rodent pancreas is an irregular-shaped thin layer of soft tissue spread throughout the upper abdomen (16). The periodic motion of the pancreas due to respiration, cardiovascular pulsations, and gastrointestinal peristalsis are challenges for obtaining high-resolution images of the organ (20). Due to these limitations, post-mortem imaging by MRI and computed tomography (CT) using GadoIodo‐EB, a contrast agent containing GdDOTA, iomeprol, and Evans blue, has been used to assess β-cells (16). Similarly, whole-fixed mice have been used to obtain 3D images of the pancreas by micro-MRI (21) but such approaches are not applicable for studying metabolic disorders *in vivo* longitudinally. The concept of using Zn^2+^ secretion as a biomarker of β-cell function was first demonstrated in mice using GdDOTA-diBPEN, a Zn^2+^-responsive MRI contrast agent with a high affinity for Zn^2+^ ions (K_D_ = 33.6 nM) (18). Upon co-secretion of Zn^2+^ and insulin from β-cells, excess Zn^2+^ ions bind in the extended side-arms of GdDOTA-diBPEN, and this initiates formation of a GdL_x_*Zn^2+^*HSA ternary complex, a slowing of molecular rotation, an increase in r_1_, and subsequently, image contrast enhancement in those tissue regions having higher amounts of free Zn^2+^ ions. Although these earlier studies (18, 19) did show pancreas image enhancement only after the mice were given a bolus of glucose to initiate GSIS, the image resolution was not sufficient to detect individual islets. In a subsequent study, an abdominal window was used to hold the tail of the mouse pancreas into a relatively fixed position and, in this case, localized regions of greater image intensity referred to as “hot spots” were found to be dispersed throughout the pancreas tail (20). Nevertheless, the relationship between the hot spots and islets releasing both insulin and Zn^2+^ could not be clearly delineated. This is an important point because it has been reported that only a small subset of islets (so-called “first responders”) release a majority of their insulin in response to a glucose challenge, while the remaining islets serve as a reserve pool of insulin (22, 23). Thus, the relationship of pancreatic “hot spots” and first responder islets remains an important question.

Although the optical window technique allowed identification of individual hot spots by MRI, this method is invasive and cannot be used for longitudinal investigations. In the current study, we demonstrate the combined use of a 2D MRI fat-saturation method, respiratory gating, and a lower affinity, Zn^2+^-responsive agent, GdL_2_ (20), allows detection of functional hot spots in the pancreas of anesthetized rats. This potentially opens the door for longitudinal studies of β-cell function *in vivo* during progression of T2DM in common rodent models of diabetes.

## Results

### *In Vivo* Detection of Zn^2+^ Secretion From the Rat Pancreas After Administration of Glucose

In a previous glucose-stimulated zinc secretion (GSZS) imaging study of the mouse pancreas, two different Zn^2+^ responsive MRI-sensors were compared, one with high Zn^2+^ binding affinity (nM, GdL_1_) versus one with lower Zn^2+^ binding affinity (µM, GdL_2_). That comparison showed that the lower affinity agent, GdL_2_, was more effective in detecting small high signal intensity pixels referred to as “hot spots” above background signal enhancement compared to GdL_1_ which produces a higher background signal (20). Given that freely available Zn^2+^ is present in all extracellular spaces of the pancreas prior to delivery of a Zn^2+^ sensitive contrast agent, one would anticipate detecting uniform enhancement of the pancreas after injection of either agent due to rapid formation of a GdL_x_*Zn^2+^*HSA ternary complex, with the contrast enhancement reflecting the amount of freely available, extracellular Zn^2+^ in the tissue before injection of the agent plus glucose. Any additional Zn^2+^ released along with insulin from β-cells should then, in principle, produce additional contrast enhancement only in those regions containing functional pancreatic islets. Our ability to detect those higher intensity regions as “hot spots” is likely then proportional to the difference between in the amount of GdL_2_*Zn^2+^*HSA formed before *versus* after GSZS. The conclusion of that prior comparative study was that GdL_2_ was more sensitive for detecting these differences. Hence, in the current rat study, we used GdL_2_ for detection of GSZS.

Sprague-Dawley (SD) rats aged 11-13 weeks were used for all imaging experiments carried out under IACUC institutional compliance. A tail-vein catheter was inserted and the animal placed in the magnet for shimming prior to injection. After the animal was ready for imaging, a bolus of either saline (500-600 uL, equivalent to twice the weight of each rat in grams, n=6) or glucose (2.75 mmol/kg from a 50% dextrose solution, n=10) was injected followed by a second bolus injection of GdL_2_ (0.1 mmol/kg from a 100 mM stock solution). Serial MRI acquisitions were collected at times 0 (pre-injection), 5, 10, 15, 20, 25, 30 min post-injection. Details of the imaging protocol and parameters are described under methods.

**Figure 1:**
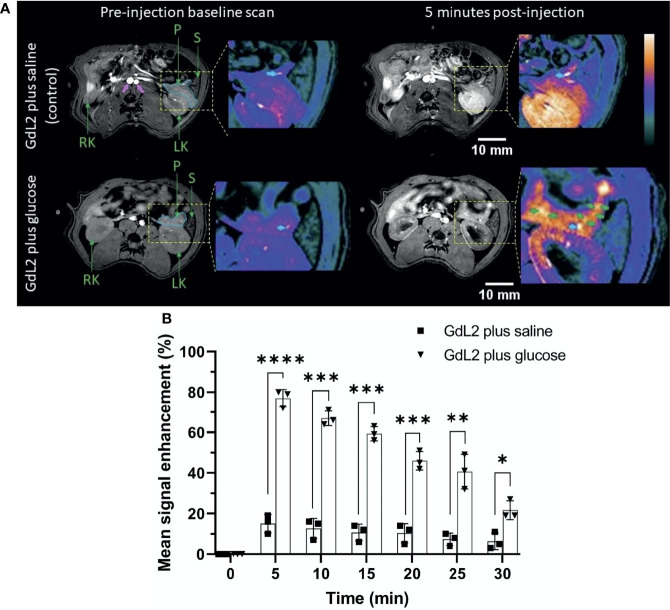
**(A)** Axial T_1_-weighted pre- and 5 min post-contrast MRI of rat pancreas after administration of GdL_2_ plus saline (top row) or GdL_2_ plus glucose (bottom row). The pancreas is outlined with solid cyan in pre-contrast images. The pancreatic tail, left kidney, and major blood vessels (pink arrows) are enhanced post-injection. The cyan arrow represents the splenic vein. [P: pancreas, S: spleen, LK: left kidney, RT: right kidney]. **(B)** Pancreas signal enhancement over time in rats injected with either GdL_2_ plus glucose or GdL_2_ plus saline. Rats injected with GdL_2_ plus glucose display significantly higher signal enhancement across all time points (p < 0.0001) compared to rats injected with GdL_2_ plus saline (p values varied from *p < 0.05, **p < 0.01, ***p < 0.001, ****p < 0.0001). The individual data points in **(B)** are shown as the mean ± SD.

For the detection of pancreatic β-cells, we focused on the pancreatic tail because of the larger number of islets in this region (23) and its clear demarcation from surrounding tissues (16). The pancreas is interspersed in the upper abdomen lateral to the spleen and left kidney (**Figure 1A**) (20). The pancreas was diffusely and weakly enhanced in the saline group (15 ± 2.2%) at 5 minutes post-injection calculated by comparing pre and post-contrast mean signal intensity of the outlined portions of the pancreas in **Figure 1A**. An intense bright spot, marked by the cyan colored arrow, reflects the splenic vein, which merges into the hepatic portal vein (16, 24). Coronal slices are shown in **Figure S1**.

Unlike in the saline group, injection of GdL_2_ plus glucose resulted in substantially higher signal enhancement in the pancreas tail (77 ± 2.1% p < 0.0001) (**Figure 1B**). Given that GdL_2_ likely does not enter cells, the higher signal enhancement observed after injection of glucose can only be attributed to release of Zn^2+^ ions from islet β-cells. Multiple focal spots of greater signal enhancement, previously referred to as “hot spots” (green arrows in **Figure 1A**) thought to represent focal clusters of β-cells, were readily detected only in animals after co-administration of GdL_2_ plus glucose. These hot spots were non-uniformly distributed throughout the pancreas tail similar to that previously detected in mice (20) and in non-human primate (25). Further images can be found in supplemental materials as **Figures S2**–**S4**. Delayed kidney enhancement was also quite apparent after injection of GdL_2_ plus glucose versus GdL_2_ plus saline (**Figure S5**). These differences likely reflect the fact that any Zn^2+^ in plasma and extracellular space immediately forms a ternary GdL_2_-Zn^2+^-albumin complex which results in slower kidney filtration of GdL_2_.

### Co-Localization of MRI Detected Hot Spots With Immunohistochemical Staining

**Figure 2:**
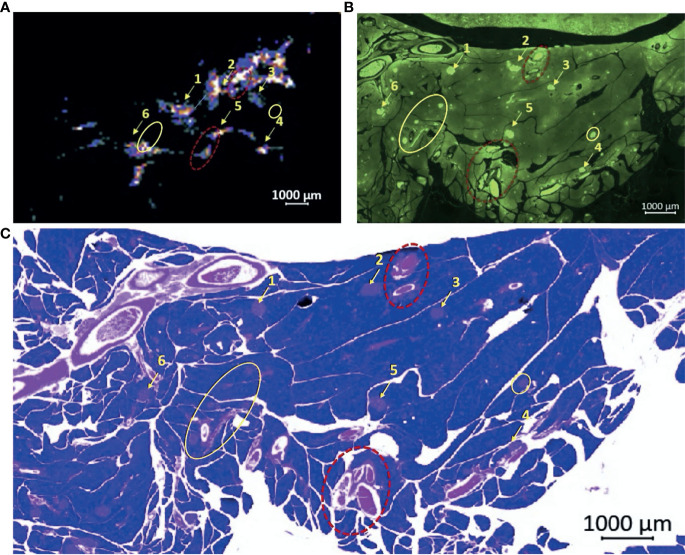
Co-registration experiment demonstrating the correlation of *ex vivo* MRI “hot spots” with islets as detected by immunohistochemical staining for insulin. **(A)** MR signals detected in an *ex vivo* pancreas removed from a rat 5 min after injection of GdL_2_ plus glucose. Setting the color scale to “cool” allows visualization of individual hot spots as indicated by the numbered arrows. **(B)** Islets identified by immunofluorescence staining for insulin, matched to the MR imaging plane shown in **(A)** demonstrates a majority of visualized “hot spots” in the MR images correspond to islets. **(C)** H&E staining also identifies islets and blood vessels. Note that some hot spots observed by MRI actually reflect blood vessels (red dotted circles), respectively. Interestingly, some insulin-stained islets do not appear as hot spots in the MR image shown in **(A)** (yellow circles).

To confirm that the observed “hot spots” correspond to islets and not blood vessels, we conducted co-registration experiments using immunohistochemical staining for insulin plus standard H&E stains. A single rat was anesthetized with isoflurane, a tail vein catheter was inserted, and a surgical laparotomy was performed. The pancreas was externalized by freeing the pancreas from the surrounding mesentery and small intestine without sacrificing the pancreatic vasculature or ligating the duodenum. GdL_2_ plus glucose was injected as described above and, after 5 minutes, the pancreas was completely excised, placed in a petri dish containing saline to maintain moisture, then imaged by MRI. After collecting images of multiple slices through the tissue, the pancreas was fixed in 10% paraformaldehyde for 48 h, then sliced and prepared for insulin-specific immunohistochemical and H&E staining. By immediately fixing the pancreas after MR, the exact location of hot spots (Zn^2+^ secretion), insulin (islets), and blood vessels (H&E) could be displayed in nearly identical anatomic planes (**Figure 2**). It should be noted however that MR imaging plane was 1 mm thick whereas the histochemical slices were only 10 μm thick so exact alignment was not certain. Nonetheless, several landmark structures (islets, blood vessels, and other identifiable tissue structures) in multiple histochemical slices co-aligned with similar structures identified in the single MRI slice. A careful examination of these images show that some hot spots (**Figure 2A**) overlap perfectly with islets identified by immunofluorescence staining for insulin (**Figure 2B**) while some hot spots correspond to blood vessels (**Figures 2B, C**, red dashed circle). This observation is not surprising because release of Zn^2+^ from β-cells and other secretory cells including prostate epithelial cells (26) should increase plasma levels of GdL_2_*Zn^2+^*HSA as well. Interestingly, the pancreatic blood vessels did not show a similar level of enhancement in animals injected with GdL_2_ followed by saline. This indicates that the enhancement seen in blood in the GdL_2_ plus glucose group must largely reflect formation of GdL_2_*Zn^2+^*HSA and not GdL_2_ alone. Additionally, there were several islets that displayed strong insulin staining but little enhancement by MRI (**Figures 2A, B**, yellow circle). This could reflect differences in the slice thickness of MRI (1 mm) versus histology (10 μM) where some of the smaller islets detected by histology may simply not be detected by MRI. Alternatively, the hot spots detected here by MRI may reflect “first responder” islets as reported previously (20) while the islets that do not enhance may reflect islets serving as a reserve pool of insulin.

### Comparisons of Functional Islets as Detected by MRI versus Total Islet Mass

**Figure 3:**
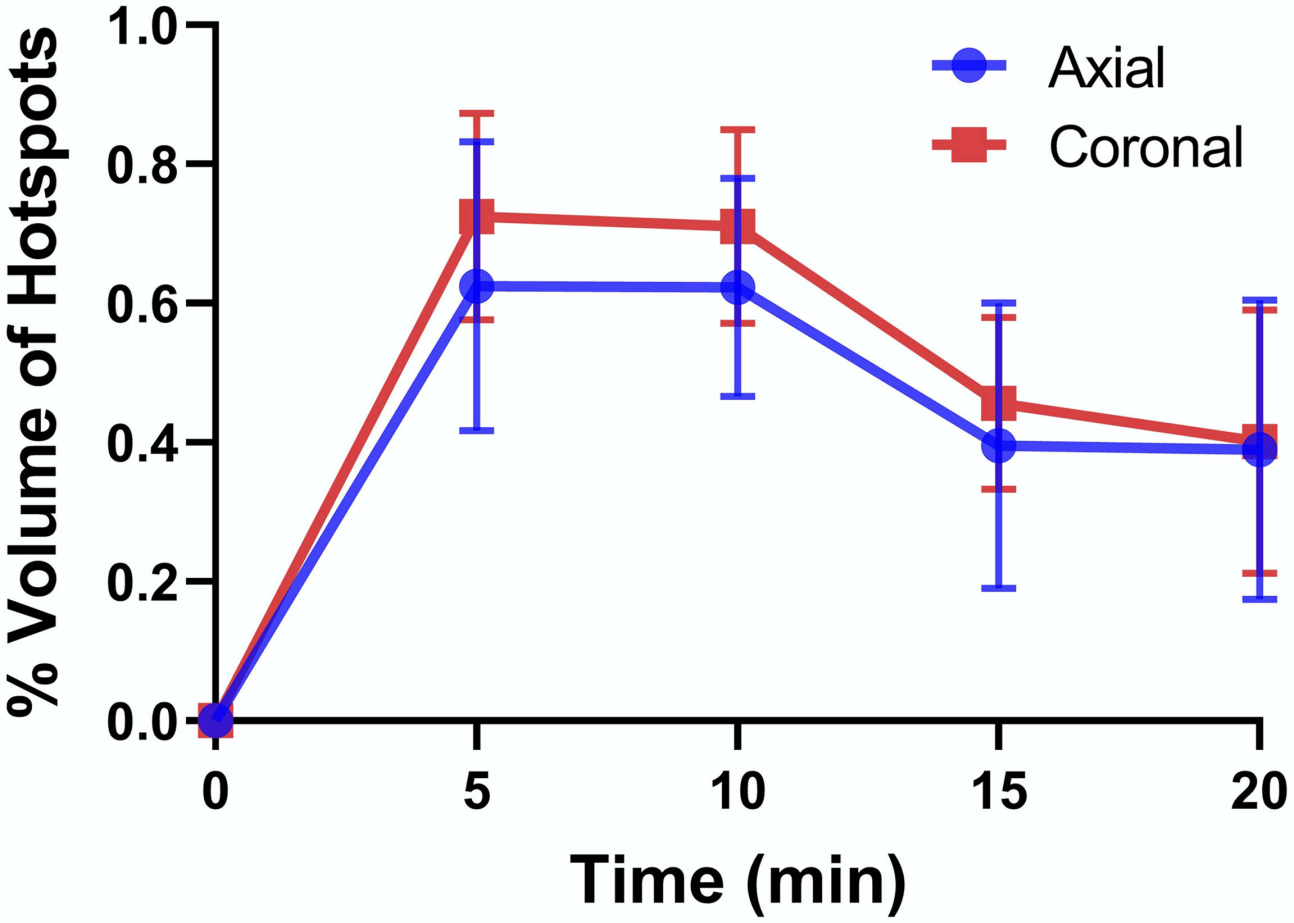
Percent volume of hot spots relative to total volume of the pancreatic tail as detected in axial and coronal slices. The data at each time point are averages ± SD for n = 3.

To examine whether volume of the hot spots detected by MRI (functional islets) correlate with published values of islet mass, the total volume of the pancreas tail was first estimated by drawing an ROI around the clearly identified pancreas tissue in each slice and multiplied by the slice thickness (1 mm) to obtain the summed volume of the pancreas tail, at least that portion detected by MRI. Similarly, the total volume of each hot spot detected in each slice was summed and this value divided by the total pancreas volume to give the total functional volume expressed as a percentage. These percentages estimated in images collected at 5 min were 0.62% and 0.73% in axial and coronal slices, respectively (**Figure 3**). Given that pancreatic islets reportedly contribute ~1-2% of the weight of the pancreas (27), these estimates are consistent with the histology data reported above that not all islets were actively releasing insulin and Zn^2+^ during the time these images were collected.

### Pharmacological Interventions Demonstrate the MRI Hot Spots Reflect Islet Function

**Figure 4:**
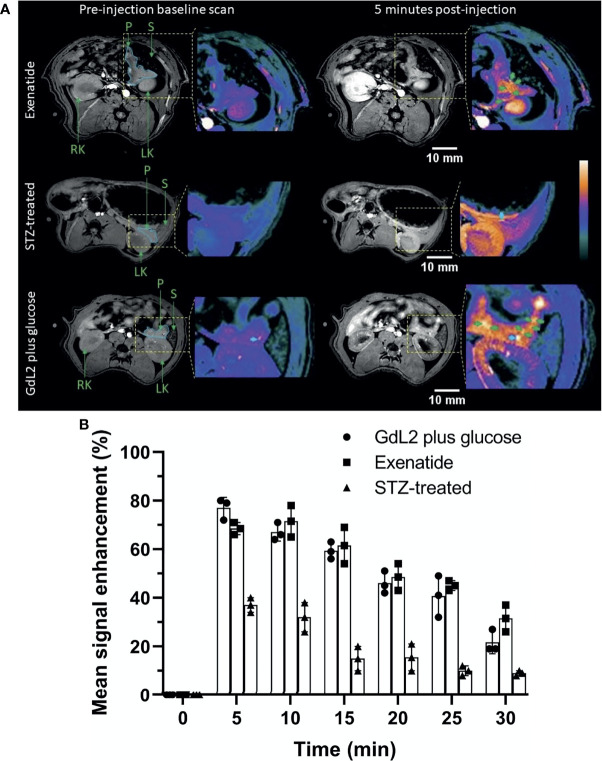
**(A)** Representative MRI images of exenatide and STZ-treated rats. Exenatide (top row) results in sustained pancreas tail enhancement with focal hot spots. In contrast, rats treated with STZ showed significantly reduced signal enhancement in the pancreas tail and no hot spots. **(B)** Comparison of pancreatic tail signal enhancement (SE) in control rats, STZ-treated rats (Type 1 diabetes model), and exenatide-treated rats. All rats were given the same dose of GdL_2_ (0.1 mmol/kg) and glucose (2.75 mmol/kg dextrose) prior to imaging. STZ results in significant reduction in SE compared to control and exenatide groups. The individual data points in **(B)** are shown as the mean ± SD.

The co-registration experiment demonstrates that GdL_2_ is capable of detecting functional islets as defined by those that release significant amounts of Zn^2+^ during secretion of insulin. To demonstrate that this method could potentially be used to monitor and assess pancreatic function in diabetic animal models, a group of rats were treated with streptozotocin (STZ, single dose, 50 mg/kg) prior to imaging (details provided in **Figure S6A**). STZ causes destruction of β-cells *via* breakage of DNA strands and activation of poly(ADP-ribose) synthesis which depletes nicotinamide adenine dinucleotide (NAD) in β-cells and hence renders rats diabetic (28). This model, although not without criticism, is often used as a model of Type 1 diabetes (29). Diabetes was confirmed by measurements of fasting blood glucose (**Figure S6B**). A second group of healthy, non-STZ treated rats were administered GdL_2_ plus glucose followed by exenatide to evaluate whether augmentation of insulin secretion can be detected by MRI. Exenatide is a glucagon-like peptide-1 receptor (GLP-1R) agonist which improves glucose homeostasis by mimicking the actions of natural GLP-1 in β-cells (30), augmenting insulin secretion in a glucose-dependent manner (31). Since the insulinotropic effect of exenatide is suppressed as the blood glucose level approaches 4 mmol/L (72 mg/dL) (32), exenatide was administered (4 nmol/kg) only in overnight-fasted rats with blood glucose levels < 70 mg/dL. Axial images of the STZ-treated rats and the exenatide-treated rats are displayed in **Figures 4A** and **S7**.

Hot spots were not evident in the pancreas in STZ-treated rats, consistent with loss of β-cell function. Nonetheless, images of the STZ-treated rat pancreas did display significantly higher signal enhancement (p < 0.05) after injection of GdL_2_ plus glucose (37 ± 2.5%) versus GdL_2_ plus saline (15 ± 2.2%). This could reflect either residual endocrine function or that Zn^2+^ released from other tissues stimulated by glucose may be sufficient to enhance the pancreas above control levels. Agent clearance from the pancreas was evident in images collected at 10 min post injection in both STZ-treated and control animals (**Figure 4B**) but, interestingly, peak signal enhancement trended to be lower at 5 min (68 ± 1.2%) compared to 10 min (72 ± 3.1%) post-injection in extenatide-treated animals (**Figure 4B**). This trend is consistent with delayed yet augmented insulin secretion in extenatide-treated animals.

### Pancreatic Hot Spot Comparisons in Control Versus Extenatide-Treated Animals

**Figure 5:**
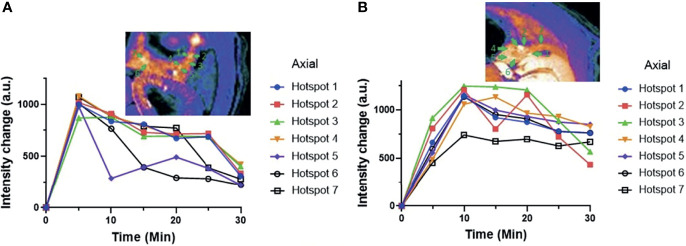
**(A)** MR signal intensities of individual pancreatic hot spots in control rats versus **(B)** exenatide treated rats over time. These data are consistent with sustained insulin secretion in animals treated with exenatide.

Previous studies have shown that some pancreatic islets act as “early responders” or islets that release insulin more rapidly than adjacent islets when stimulated by glucose (33). To investigate this further, the signal intensity within individual hot spots in both the exenatide and control (GdL_2_ plus glucose only) groups are compared (**Figure 5**). In the glucose control group (not treated with exenatide), the signal intensity within each of the identified hot spots decreased steadily with time after 5 min. However, the rate of decline was uneven with signal enhancement decaying rapidly in some hot spots even in the 10 min image (i.e. axial hotspot #5 in **Figure 5A**) while others maintained high signal enhancement even in 20 and 25 min images (i.e. hotspots 1–4 in **Figure 5A**). In comparison, not only did the signal intensity of all hot spots increase between 5 and 10 min, but the rate of signal intensity decrease was also more gradual at extended times compared to control (**Figure 5B**) and clearance of plasma glucose was more rapid (**Figure S8A**) consistent with prolonged release of insulin over the 30 min period.

## Discussion

A rigorous, non-invasive method of imaging pancreatic islet function *in vivo* could aid in substantially improving our understanding of the pathogenesis of diabetes mellitus as well as offer a method to evaluate the impact of anti-diabetes drugs in real-time. While rodent models are widely used in preclinical studies, there are many technical challenges in distinguishing the pancreas from adjacent organs by MRI including respiratory/cardiovascular motion (34), the interspersed, poorly defined anatomy of the pancreas, and the lower-resolution offered by 3D imaging (16). In this study, we present a 2D MRI sequence combined with the use of a Zn^2+^-responsive contrast agent, GdL_2_, to enable real-time imaging of insulin secretion from rat pancreatic β-cells *in vivo*. This fat-suppressed, signal-averaged protocol yielded high-resolution 2D images of the pancreas and respiratory gating further minimized artifacts and improved overall image quality.

Major secretory tissues like pancreas, prostate, and mammary glands contain abundant Zn^2+^ (35). Thus, Zn^2+^ has received considerable attention in the field of biomedical imaging, resulting in the development of both fluorescence (26, 36) and MRI probes (19, 20, 37). MRI contrast agents that turn on in the presence of high Zn^2+^ allow indirect detection of insulin secretion as Zn^2+^ ions are co-secreted into interstitial spaces along with insulin. Interestingly, Zn^2+^ secretion was detected as punctate, enhanced “hot spots” predominantly in the tail of the rat pancreas only after administration of glucose to stimulate insulin secretion. Similar enhanced hot spots were detected in the mouse pancreas in prior studies but only after the tail was fixed in position by use of an abdominal window (20) and, more recently, in the pancreas of non-human primates after infusion of a similar Zn^2+^-sensitive agent (25). While the hot spots detected in these previous studies were assumed to reflect individual islets or clusters of islets, co-registration of immunohistochemical stained islets and the MRI hot spots in the current study showed near perfect concordance between the MRI data and histology. Other MRI methods, such as MnCl_2_-enhanced MRI, do reflect overall β-cell function in the pancreas *in vivo* (24) but can only detect individual islets in the exteriorized mouse pancreas (38). The GSZS method described here offers the advantage of imaging β-cell function in individual islets in the rat pancreas *in vivo* and is potentially applicable to longitudinal studies.

What do these enhanced hot spots actually reflect? A close analysis of the co-registration experiment (**Figure 2**) reveals that while most of hot spots correspond to individual islets, some also reflect blood vessels. In contrast, no hot spots reflecting either islets or blood vessels, were detected in MR images of the saline control group. This is not entirely unexpected since insulin must be secreted into the circulatory system to have systemic effects. If the relaxivity of GdL_2_ alone was sufficient to cause blood vessel enhancement, we would expect to see hot spots in images after injection of GdL_2_ with either saline or glucose. Since this is not the case, we conclude the blood vessels detected in images of the glucose group must reflect formation of GdL_2_*Zn^2+^*HSA in blood after secretion of insulin and Zn^2+^ from pancreatic β-cells and from other zinc-secreting tissues. Nullifying the blood pool signal using MRI sequences such as black-blood imaging (39) could selectively suppress the signal intensity in blood vessels and thereby increase the specificity of GdL_2_ and Zn^2+^ secretion only from functioning pancreatic islets.

The response of β-cells to changes in glucose is known to be quite heterogeneous with some cells responding rapidly and strongly while others remain relatively quiescent. The molecular mechanisms underlying these differences were nicely summarized in a recent review article (40). Even within individual islets, it has been shown that subpopulations of β-cells control islet calcium levels and insulin release dynamics in adjacent cells (33). In the context of the current study, Li, et al. have also shown using an extracellular zinc-responsive fluorescence sensor that secretion of insulin (as detected by Zn^2+^ release) is highly coordinated among β-cells in an single islet (36). Studies of islets *in vitro* have shown they are more or less functionally identical and that all islets contribute small amounts of insulin in response to an increase in glucose (41). However, this does not appear to be true *in vivo* where it has been shown that a few select islets act as “first responders” by secreting insulin most rapidly after exposure to rising levels of glucose while other islets act as a slower release, reserve pool of insulin (22). The imaging observations reported here certainly do not definitively identify the observed hot spots as first responder islets but the fact that the entire pancreas, or at least those regions of pancreas known to contain more islets, is not uniformly enhanced during GSIS is at least consistent with this hypothesis. As been demonstrated, some β-cells initially release only a portion of their insulin granules in response to glucose, the readily releasable pool (RRP), while a second pool of granules are released more slowly during a more prolonged second phase of insulin secretion (22, 42). The observation that some insulin-stained islets were not detected as “functioning” islets by MRI further supports though does not prove this hypothesis. It has been estimated that ~0.05% of total insulin stores is released from the pancreas per min when glucose rises to 15 mM (43) so our observation that only 0.6- 0.7% of the total islets in the rat pancreas tail appeared as hot spots during each 5 min MRI data collection is consistent with these previous observations.

A previous report showed that GLP-1 augments first phase insulin secretion between 5 to 10 min after injection of 10 μg/kg of GLP-1 and 0.5 g/kg glucose in fasted Sprague Dawly (SD) rats (44). Similarly, the hot spots detected in this study reached a near-identical maximum signal enhancement 5 min post injection of GdL_2_ plus glucose in untreated rat while those animals pre-treated with exenatide show a maximum signal enhancement closer to 10 min post injection. This indicates that either all islets release a similar amount of insulin and Zn^2+^ or that GdL_2_ becomes saturated with Zn^2+^ thereby limiting the amount of GdL_2_*Zn^2+^*HSA that can be formed. A comparatively higher and prolonged signal enhancement in animals treated with exenatide (**Figure 4B**) is consistent with augmented insulin secretion in those animals. The more rapid decrease in plasma glucose in the exenatide-treated animals also supports augmented insulin/Zn^2+^ secretion in those animals (**Figure S8**).

A previous imaging study of the non-human primate pancreas using a different Zn^2+^-sensitive agent (CP-027) showed higher levels of insulin and C-peptide in blood plasma after injection of CP-027 plus glucose compared to a non-Zn^2+^ binding control agent plus glucose (25). This interesting observation suggests that CP-027 itself may augment insulin secretion by reducing plasma levels of free Zn^2+^ but one major difference in that study was that the agent was infused at a continuous rate throughout the imaging experiment. CP-027 also has an ~10^3^ fold higher affinity for Zn^2+^ compared to GdL_2_ so it is unlikely that GdL_2_ had a similar augmentation effect in the current study. Indeed, as shown in **Figure S8B**, plasma levels of Zn^2+^ did not increase significantly in any group of animals included in this study after infusion of GdL_2_.

The absence of hot spots in the pancreatic tail of STZ-treated diabetic rats (**Figure 4A**) confirms that the hotspots detected in the pancreatic tail of normal control rats and exenatide-treated rats (**Figure 4**) indeed reflect β-cell function. Our estimate of the volume fraction of functional islets (0.62- 0.73% of the pancreas tail, **Figure 3**) was less than the reported fraction of islet mass (1–2%) (17) and entirely consistent with the histology data showing that many islets are present in the tail that were not enhanced in MR images. This interesting observation suggests that the β-cells in those islets that appear not to be releasing insulin under these experimental conditions could perhaps be stimulated to release insulin either with a more prolonged infusion of glucose or addition of other secretagogues.

In summary, this study was devoted to the non-invasive imaging of pancreatic β-cell function in rats *in vivo* by using of optimized 2D MRI methods and the Zn^2+^ sensor, GdL_2_. Clearly discernible signal-enhanced hot spots were identified as functional islets in the tail of the pancreas. We believe this imaging method will be useful for monitoring the efficacy of new drugs designed to stimulate β-cell function and, given that it is non-invasive and allows for longitudinal studies, for monitoring progressive loss of β-cell function during development of type 2 diabetes in rodents.

## Methods

### Preparation of GdL_2_ Stock Solutions

GdL_2_ was synthesized, purified, and characterized as described previously (20). A stock solution containing 100 mM GdL_2_ dissolved in 100 mM Tris buffer, pH = 7.4 was used in all *in vivo* MRI experiments.

### Animals

A total of 24 rats (male, 11–14 weeks age; Sprague Dawley #400, Charles River Laboratories, Wilmington, MA) were used for *in vivo* MR imaging experiments. 6 rats were imaged after tail vein injection of GdL_2_ plus saline, 6 rats were imaged after injection of GdL_2_ plus glucose, 6 rats were used in the STZ-treated group and 6 rats were imaged after injection of GdL_2_ plus glucose plus exenatide.

### *In Vivo* Imaging of the Pancreas in Rat

All animal experiments were performed in accordance with guidelines set by the UT Southwestern Institutional Animal Care and Use committee (IACUC). Male Sprague Drawly rats were fasted overnight before imaging. Rats were anesthetized with isoflurane (3–4%) mixed with oxygen. The details of the imaging sequence and parameters are given in **Table 1**. The rats were secured inside 72 mm volume coil in supine position with the upper abdominal region at the isocenter of a 9.4 T Varian preclinical MRI scanner. Either 2.75 mmol/kg glucose dissolved in 0.9% normal saline (0.5 g/L solution) or 0.9% normal saline alone were injected followed immediately by 0.1 mmol/kg GdL_2_. The dose of saline was twice the gram body weight in µL. In the exenatide group, 4 nmol/kg of exenatide was injected immediately after GdL_2_ plus glucose but before imaging. All injections were intravenous and *via* a tail vein catheter. Consecutive scans were collected at 0, 5, 10, 15, 20, 25 and 30 min post-injection.

**Table 1:** MRI sequence protocol and parameters.

Sequence	Imaging plane	TR (ms)	TE (ms)	EA (α)	A	Matrix (RO X PE)	FOV (mm mm)
GEMS with fat saturation	Axial and Coronal	129.81	4.27	43°	7	256 X 256	60 X 60

Where GEMS, Gradient echo 2D multi-slice; TR, repetition time; TE, echo time; EA, Ernest Angle; A, Signal average.

Imaging data were collected between breaths using respiratory gating to minimize the blurring effects of respiratory motion. It should be noted that conventional MRI scans that apply phase-encoding (PE) and readout (RE) gradients along respective X (isocenter) and Y directions (**Figure S9A**), produce blurring and ghosting (BG) due to distortion of gradient linearity along isocenter (45, 46). This, consequently, degrades the image quality of the pancreas when a maximum area of pancreatic region coincides along isocenter during axial image acquisition (**Figure S9B**). In the present study, we altered directions of PE and RO gradients, and applied along Y and X directions respectively to minimize BG in the images (**Figures S9C, D**).

### Co-Registration of *Ex Vivo* MRI With Histology

A male Sprague Drawly rat was fasted overnight, anesthetized with isoflurane (3 ~ 4% in medical oxygen), and catheterized *via* tail vein. After the pancreas was externalized as described in the text, glucose (2.75 mmol/kg) and GdL_2_ (0.1 mmol/kg) were injected via the tail vein and 5 min post-injection, MR images were collected using identical parameters as used *in vivo*. After the *ex vivo* images were collected, the animal was sacrificed and the pancreas placed in 10% paraformaldehyde (PFA) in a super cassette (75 x 52 x 17 mm) for another 48 h and then passed into EtOH for 24 h at 4°C. After fixation, the tissue was sliced into 10 μm slices, sequentially into 3 slices as a set with a 1 mm interval (total 15 slides). The first slide of each set was stained with hematoxylin and eosin (H&E) to visualize pancreatic islets and the second slide was used for immunohistochemistry. For immunostaining, pancreatic formalin-fixed paraffin embedded (FFPE) were washed in xylene and rehydrated by wash in a series of graded EtOH (80% ~ 50%) and water. After antigen retrieval and blocking (10% donkey serum in 0.1% PBST for 1 h), the sample was stained with primary antibody (Polyclonal Guinea Pig Anti-Insulin, Dako A0564, 1:500) for 4hr at RT and secondary antibodies (donkey anti-guinea pig IgG conjugated with AF488, Jackson ImmunoResearch 706-545-148, 1:200) for 0.5 h at RT. The labeled FFPE slides were shielded with a glass coverslip using Fluoromount-G (southern Biotech) and imaged by Zeiss Axio Scan.Z1. The image processing was performed by using Image J and ZEN lite software. The H&E staining were performed by the Histopathology Core at UTSW.

### Respiratory Gating

ASA Instruments, Inc., Stony Brook, NY 11790, USA, Model: Control/Gating Module was used for respiratory gating. We collected data during breath-hold period following inspiration with short delay time of 30 ms in the respiratory-gated GEMS sequence. The respiratory rate was maintained at 32-40 breaths per minute in each rat by varying the amount of inhaled isoflurane.

### Image Analysis

The raw 32-bit image files were obtained as Varian’s.fdf files. The images were stacked and converted to.tiff files using ImageJ (imagej.nih.gov). The zoomed outlined images were processed to produce “COOL” pseudocolor images to delineate the tissues—pancreas, spleen and kidney. Normalized signal intensity was determined using standard reference (phantom) for quantitative evaluation of pancreatic signal enhancement. For the determination of signal intensity, different regions of interest (ROI) were drawn on pancreas (green area) and hotspots (pink dots) in axial and coronal sections manually to calculate mean signal intensity (*S*) on the respective tissues as shown in **Figure S10**. The prescan signal intensity (*S*) was subtracted from their respective post-scan signal *S*. The percentage change in mean signal intensity was calculated as Δ*S* % = (│Δ*S*_post_ – Δ*S*_pre_│)/Δ*S*_pre_) X 100%.

### Islet Volume Calculations

Seven axial and coronal slices (each 1 mm thick) were stacked and converted to.tiff files using Fiji (https://imagej.net/Fiji). ROIs were drawn of the pancreas tail and separately around each individual hotspot in both axial and coronal planes, on each slice that the tail or hot spots were visualized. The “segmentation editor” tool was used to determine areas of the pancreas tail and each individual hot spots. The volume of the islets and pancreas in each slice was determined by multiplying surface area by the slice thickness (1 mm). Finally, the total pancreas tail volume was determined by adding the volume of the pancreas at each individual slice, and the conglomerate hot spot volume was determined by adding the volume of each individual hot spot.

### Blood Analysis

#### Measurement of Blood Glucose Levels

12 rats (male, 11–14 weeks age; Sprague Dawley #400, Charles River Laboratories, Wilmington, MA) were fasted (only food removal) overnight. Four rats were administered either (i) GdL_2_ plus saline, (ii) GdL_2_ plus glucose, or (III) GdL_2_ plus glucose followed by exenatide at the same dose used in imaging. Blood samples were collected via a tail vein.

The blood glucose level (BGL) (in mg/dL) was measured in blood from tail-vein by using commercially available glucose meter (OneTouch^®^ Ultra^®^ 2) at 0 (pre-injection), 5 and 30 min post-injection.

#### Quantification of Zn^2+^

The Zn^2+^ concentration was quantified *via* inductively coupled plasma mass spectrometry (ICP-MS) at the University of Texas at Dallas. The sample preparation protocol for whole blood samples were slightly modified compared to a published method (47). 50µl of plasma was mixed with 100 µl of approximately 65% nitric acid and 50µl of hydrogen peroxide (H_2_O_2_) in a screw capped 15 ml polypropylene tube. The samples were heated for 2 h at 60°C after vortex until all denatured proteins were digested and a clear solution were obtained. The digestion solutions were diluted with 1.8 ml of deionized water to give a final nitric acid concentration of 5%.

### Statistical Analysis

Descriptive statistics analyses on continuous variables are presented as mean ± SD. Statistical analyses were performed using GraphPad Prism v.8 (GraphPad Software, Inc., La Jolla, CA). The statistical significance was assessed by two-way analysis of variance (ANOVA) with ‘Sidak’ *post hoc* analysis for multiple statistical comparisons. The *p* values less than 0.05 (*p<0.05, **p<0.01, ***p<0.001 and ****p<0.0001) were considered significant.

## Data Availability Statement

The raw data supporting the conclusions of this article will be made available upon request.

## Ethics Statement

The animal study was reviewed and approved by UT Southwestern Institutional Animal Care and Use Committee (IACUC).

## Author Contributions

BT, DP, EHS, and ADS designed research. BT, PK, EHS, SC, and GS performed the experiments. BT wrote the first version of manuscript. BT, PK, DP, EHS, and ADS analyzed the data. BT, EHS, PK, DP, SC, and ADS reviewed and commented on the manuscript at all stages. ADS supervised the research. All authors contributed to the article and approved the submitted version.

## Funding

The authors gratefully acknowledge the financial support from the National Institutes of Health (NIH) grant DK-095416.

## Conflict of Interest

The authors declare that the research was conducted in the absence of any commercial or financial relationships that could be construed as a potential conflict of interest.

## Publisher’s Note

All claims expressed in this article are solely those of the authors and do not necessarily represent those of their affiliated organizations, or those of the publisher, the editors and the reviewers. Any product that may be evaluated in this article, or claim that may be made by its manufacturer, is not guaranteed or endorsed by the publisher.
