# Supplementary material for: Imaging β-Cell Function Using a Zinc-Responsive MRI Contrast Agent May Identify First Responder Islets
Source: Front Endocrinol (Lausanne). 2022 Jan 31;12:809867. doi: 10.3389/fendo.2021.809867 (PMC8842654; doi:10.3389/fendo.2021.809867)
Supplement: Supplementary file 1 [file Presentation_1.pdf]

Supplementary Information

Imaging β-cell function using a zinc-responsive MRI contrast agent may identify first responder islets

Bibek Thapa,[a†] Eul Hyun Suh,[a†] Daniel Parrott,[a,b†] Pooyan Khalighinejad[a], Gaurav Sharma,[a] Sara Chirayil[a], and A. Dean Sherry[a,b,c,1]

[a]*Advanced Imaging Research Center, The University of Texas Southwestern Medical Center, Dallas, Texas 75390, United States;*

[b]*Department of Radiology, The University of Texas Southwestern Medical Center, Dallas, Texas 75390, United States;*

[c]*Department of Chemistry and Biochemistry, The University of Texas at Dallas, Richardson, Texas 75080, United States.*

[†] *These authors contributed equally to this work*

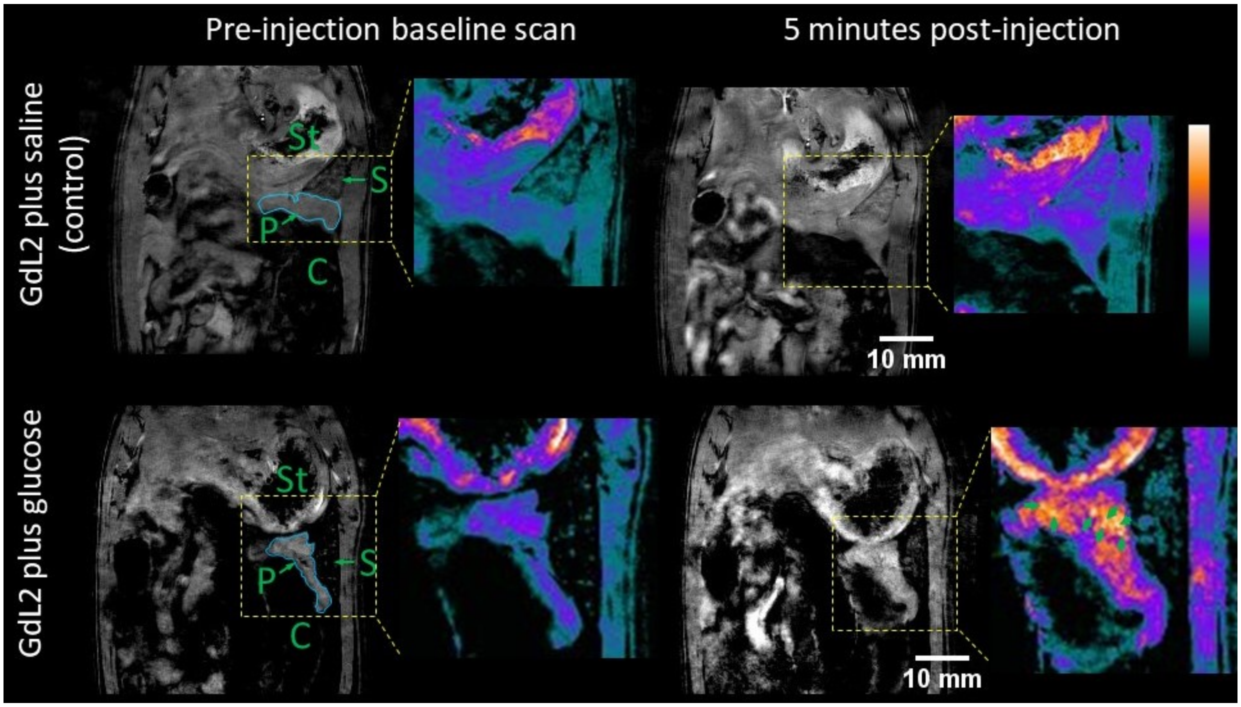

Figure S1. Coronal T_1-weighted pre- and 5 min post-contrast MRI of rat pancreas after administration of GdL_2 plus saline (top row) and GdL_2 plus glucose (bottom row). The pancreas is outlined (cyan) in the baseline images. The pancreatic hot spots are indicated by green arrows in the 5 min post-injection of GdL_2 plus glucose image (bottom right). [P: pancreas, S: spleen, St: stomach, C: caecum]

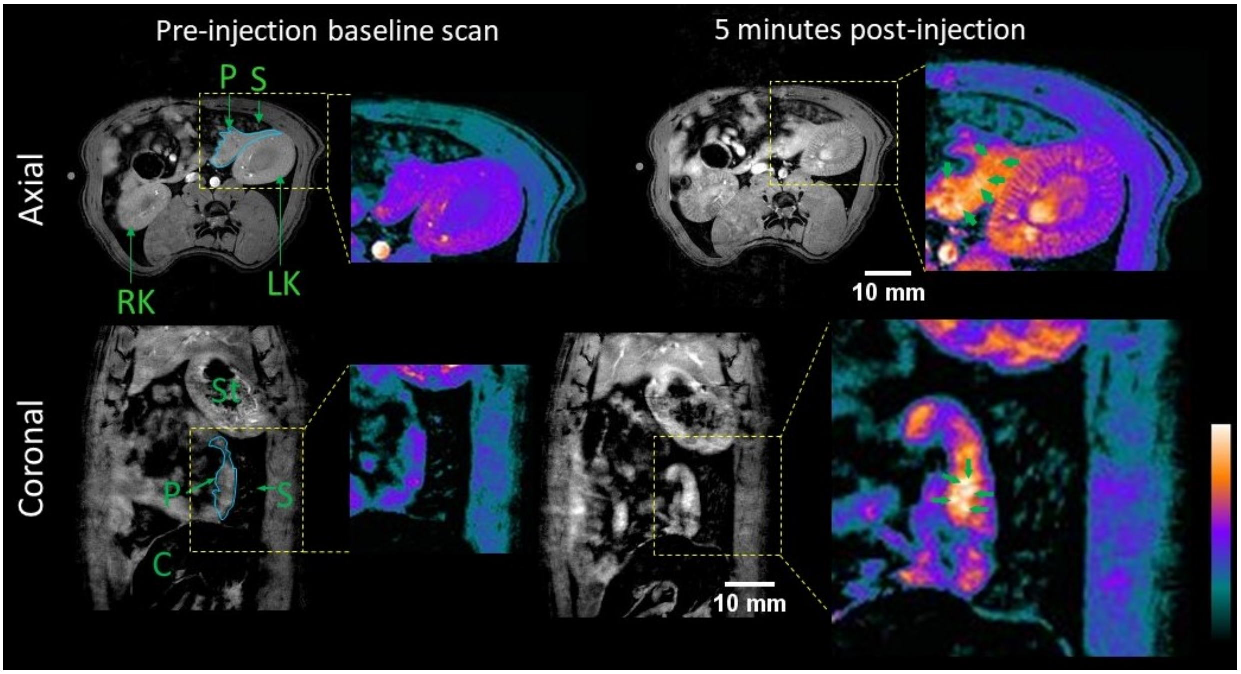

Figure S2. Axial (top row) and coronal (bottom row) T_1-weighted pre- and 5 min post-contrast MRI of the rat pancreas after administration of GdL_2 plus plus glucose. The pancreas is outlined (cyan) in the baseline images. The green arrows indicate hot spots observed in the tail of pancreas at 5 min post-injection (top right and bottom right). [P: pancreas, S: spleen, St: stomach, LK: left kidney, RT: right kidney, C: caecum]

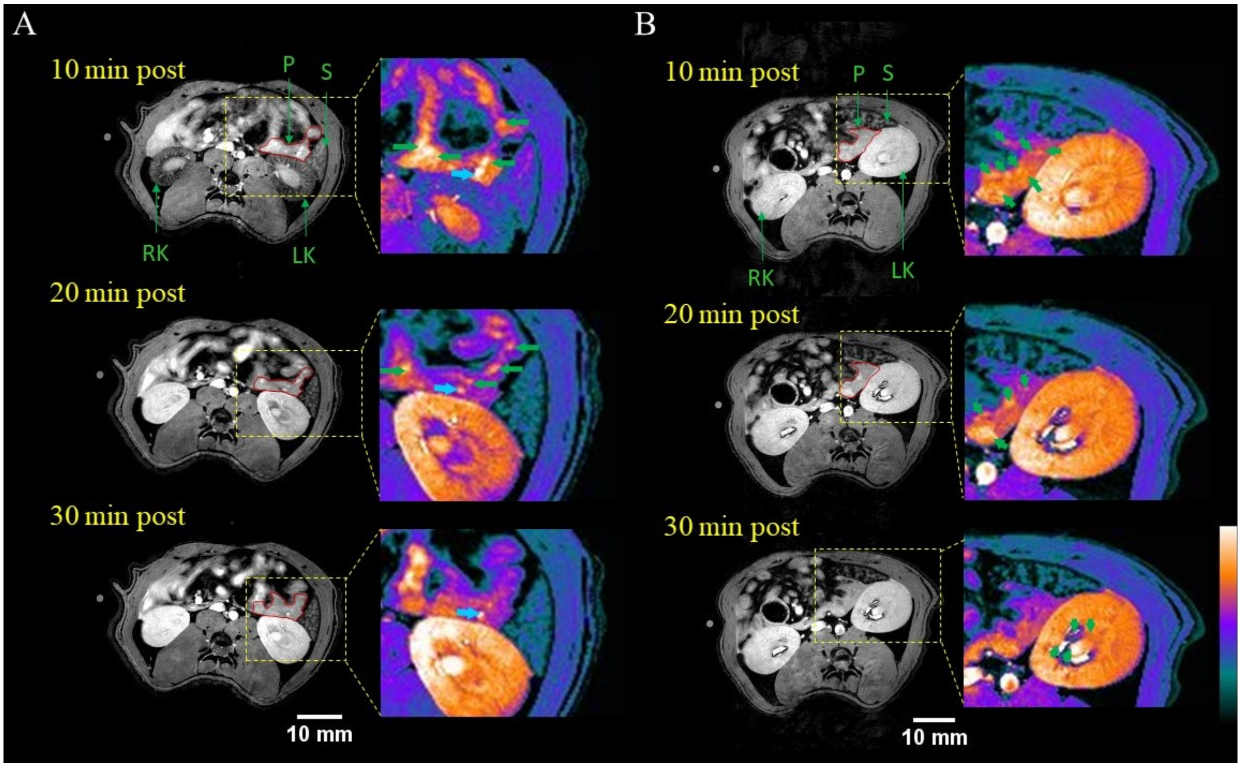

Figure S3. Temporal images of hot spots (green arrows) in axial T_1-weighted MRI of rat pancreas after administration of GdL_2 plus glucose to two different rats. The pancreas is outlined in red. Column A shows the hot spots observed at 10, 20 and 30 min post-injection in one rat (5 min post-injection image is shown in Fig. 1/bottom row) and column B shows the same timeline for a second rat (5 min post-injection image is shown in Fig. S2/Axial section). [P: pancreas, S: spleen, LK: left kidney, RT: right kidney]

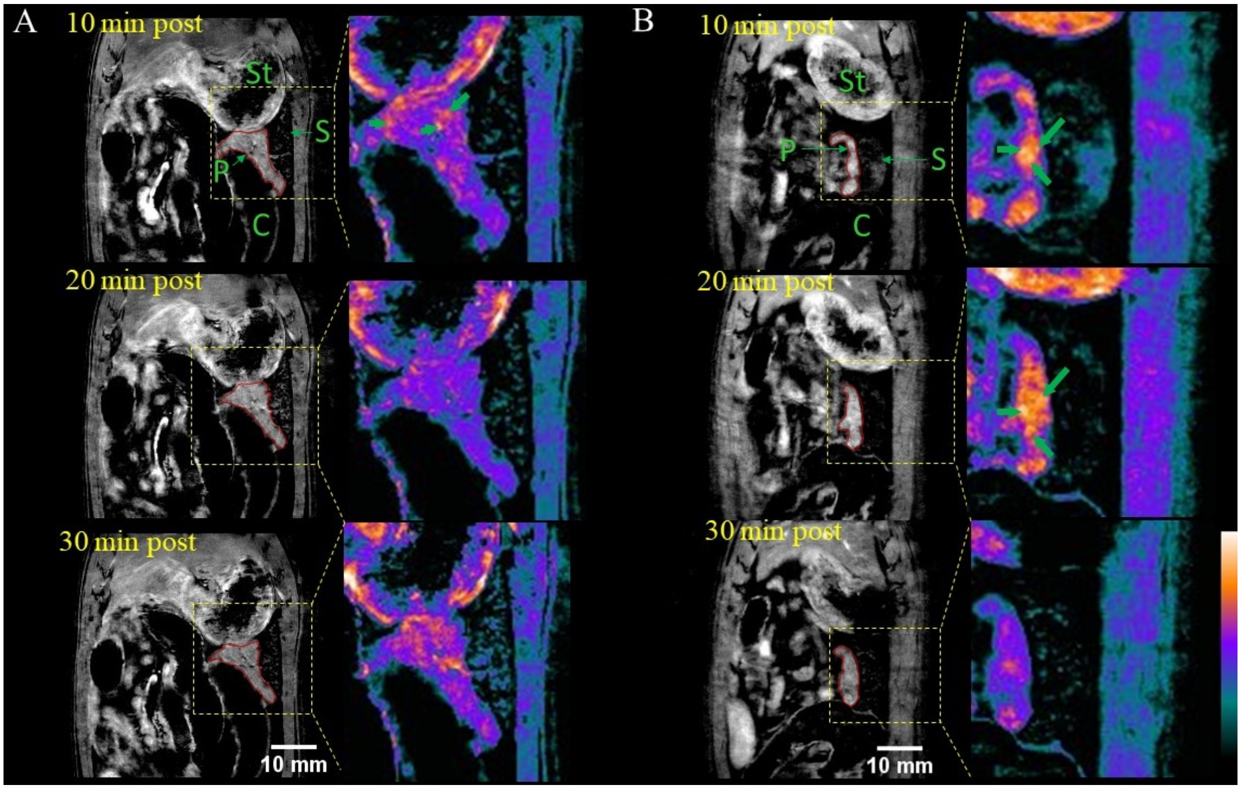

Figure S4. Temporal images of hot spots (green arrows) in coronal T_1-weighted MRI of rat pancreas after administration of GdL_2 plus glucose to two different rats. The pancreas is outlined in red. Column A shows hot spots observed at 10, 20 and 30 min post-injection in rat #1 (5 min post-injection image is shown in Fig. S1/bottom row) and column B shows hot spots over the same timeline for rat #2 (5 min post-injection image is shown in Fig. S2/Coronal section). [P: pancreas, S: spleen, St: stomach, C: caecum]

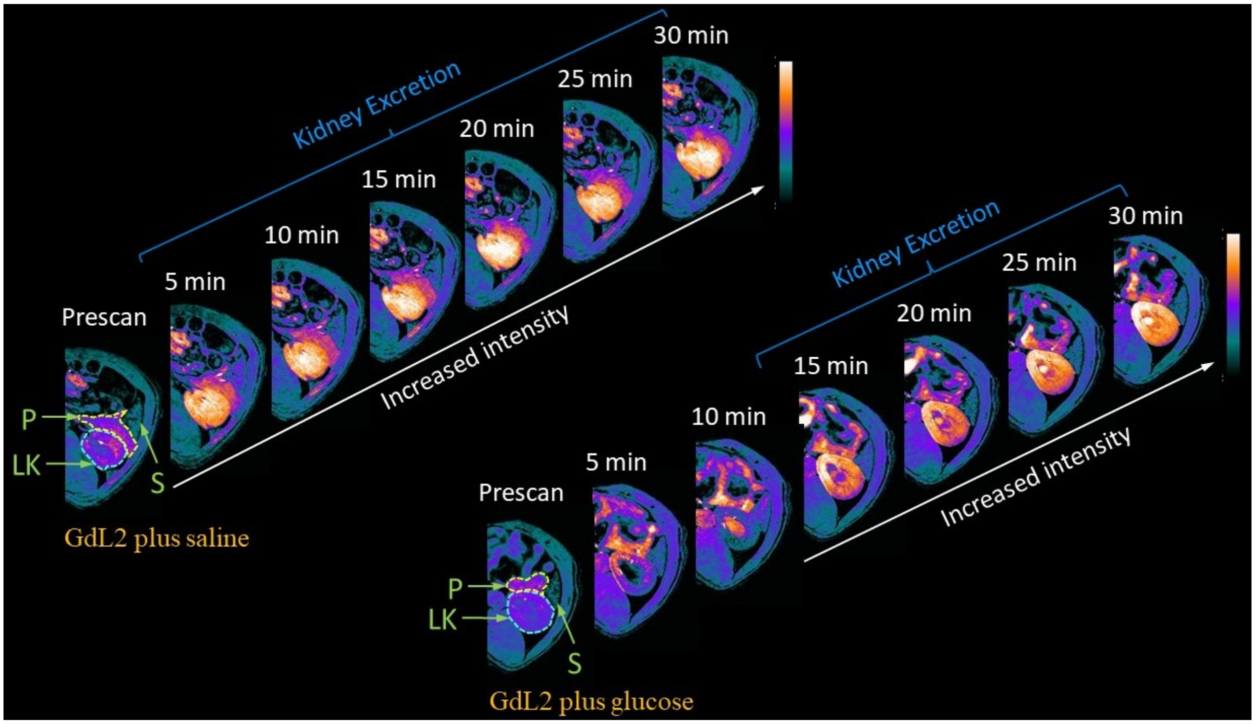

Figure S5. MRI axial sections of rat kidney obtained at 0 (prescan), 5, 10, 15, 20, 25, 30 minutes of post-injection of GdL_2 plus saline (top panel) and GdL_2 plus glucose (bottom panel). The kidney and pancreas are outlined by the dashed cyan and yellow lines, respectively, in the prescan images. [LK: left kidney, P: pancreas and S: spleen].

Streptozotocin (STZ) treatments of rats:

The STZ treatments of rats was performed according to the standards of the UT Southwestern Institutional Animal Care and Use Committee (IACUC). 8 normal Sprague Dawley (SD #400) male rats (age 9-10 weeks old) were used. A single bolus dose of 50 mg/kg of STZ in citrate buffer was administered via intraperitoneal (IP) injection. After 3 days, the rats were fasted overnight followed by the measurements of blood glucose level (BGL) from tail tip. The 4 rats were found to be hyperglycemic.

A **B**

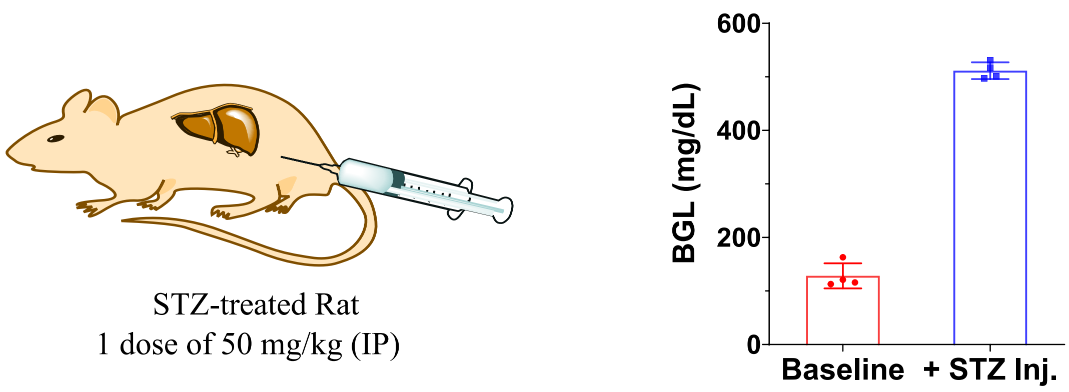

Figure S6. (**A**) IP injection of STZ in living rat. (**B**) Fasting blood glucose levels (BGLs) at pre- and post-treatment of STZ in 4 rats. Data are shown as mean ± SD

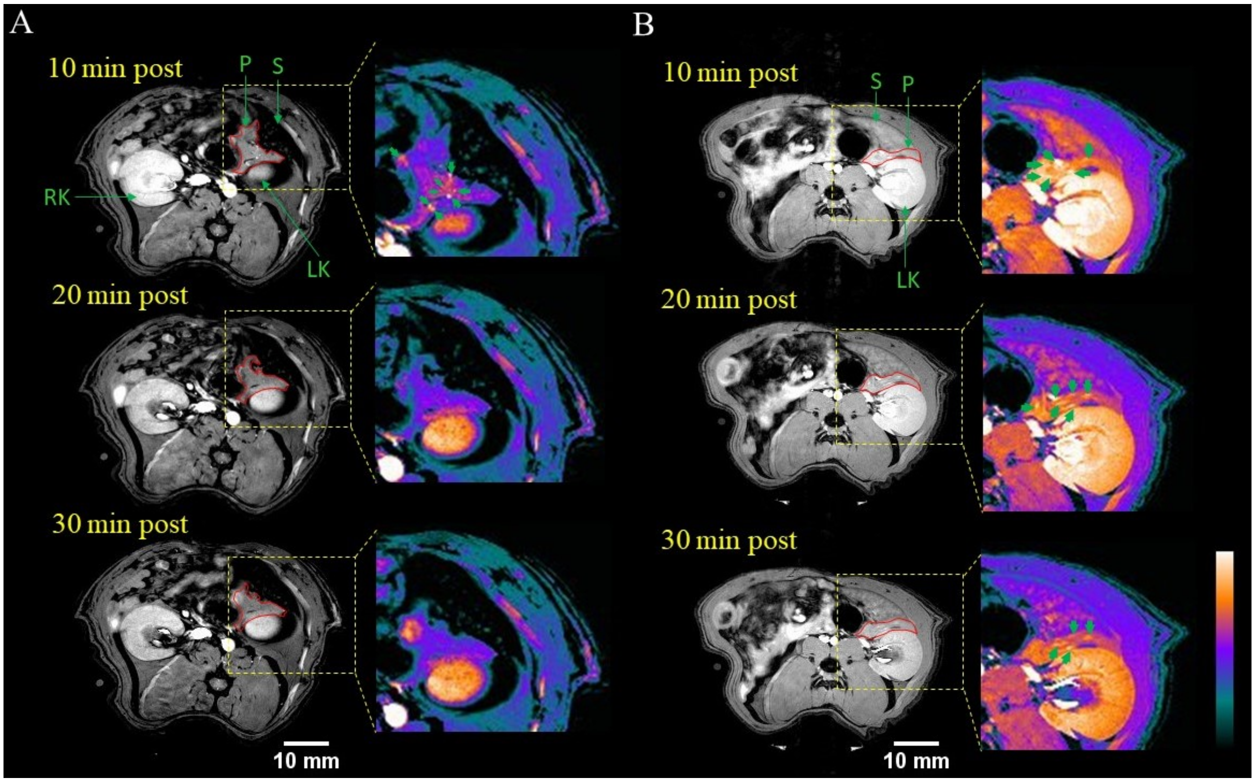

Figure S7. Temporal images of hot spots (green arrows) in axial T_1-weighted MRI of rat pancreas after administration of GdL_2 plus glucose followed by exenatide to two different rats. The pancreas is outlined in red. Column A shows pancreatic hot spots observed at 10, 20 and 30 min of post-injection in rat #1 (5 min post-injection image is shown in Fig. 5/Exenatide) while column B shows hot spots observed over the same timeline in rat #2 (5 min post-injection image is shown in Fig. 7B/inset). [P: pancreas, S: spleen, LK: left kidney, RK: right kidney]

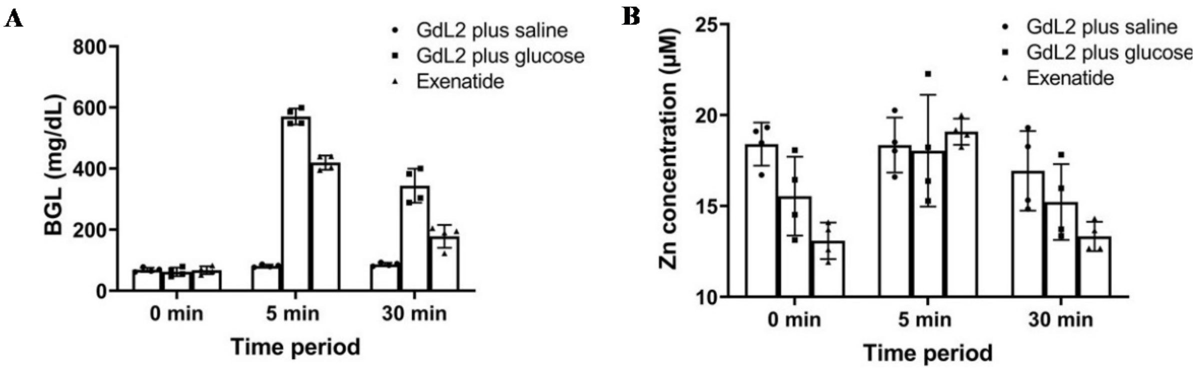

Figure S8. Blood glucose (BGL) (A) and plasma zinc concentrations (B) in rats (n = 4) after overnight fasting/pre-injection [baseline (0 min)] and post-injection (I.V.) of GdL_2 plus saline, GdL_2 plus glucose, or GdL_2 plus glucose followed by exenatide (Exenatide) at 5 min, and 30 min. The data at each time point are shown as mean ± SD.

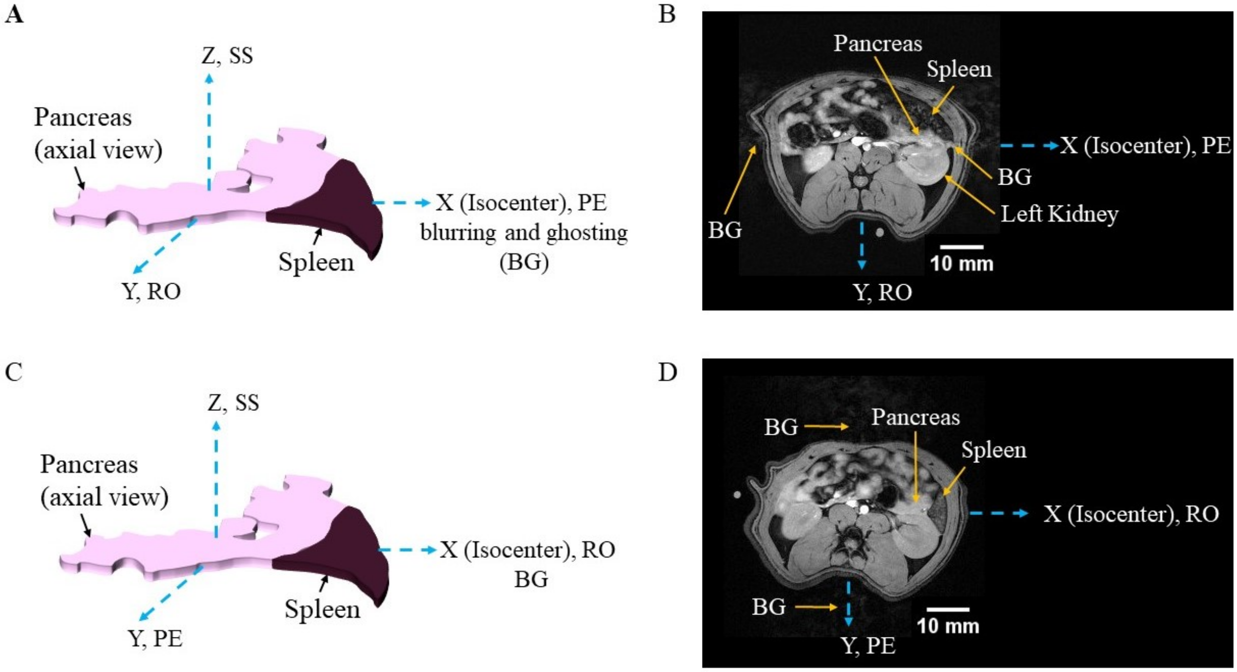

Figure S9. 3D representations of the rat pancreas and spleen showing their orientation with respect to the direction of the phase encoding (PE), readout (RO) and slice-select (SS) gradients. **A.** When the PE gradient is along the X-axis (isocenter), periodic movements from respiratory, cardiovascular pulsations and peristalsis produce blurring and ghosting (BG) of the pancreas as shown in **B**. However, when the PE gradient is along the Y-axis and the RO gradient is along the X-axis (isocenter) as illustrated in **C** (used in this study), the BG artifacts in the pancreas are minimized (image shown in **D**).

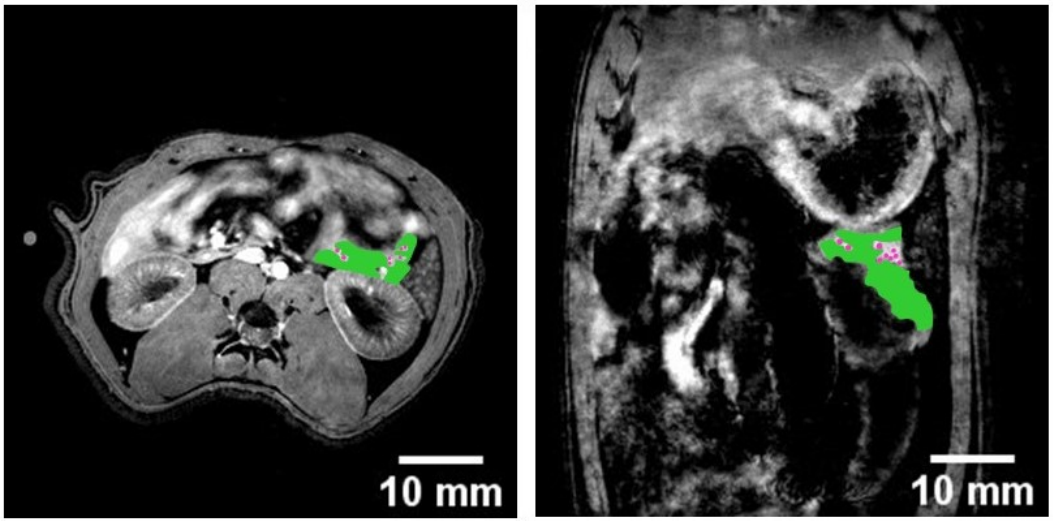

Figure S10. Illustration of typical ROIs drawn for the pancreas (green) and pancreatic hotspots (pink) for calculating signal intensities. Left image (axial), Right image (coronal).
